# Simulating water markets with transaction costs

**DOI:** 10.1002/2013WR014493

**Published:** 2014-06-06

**Authors:** Tohid Erfani, Olga Binions, Julien J Harou

**Affiliations:** 1 School of Mechanical, Aerospace and Civil Engineering, University of ManchesterManchester, UK; 2 Department of Civil, Environmental and Geomatic Engineering, University College LondonLondon, UK

## Abstract

This paper presents an optimization model to simulate short-term pair-wise spot-market trading of surface water abstraction licenses (water rights). The approach uses a node-arc multicommodity formulation that tracks individual supplier-receiver transactions in a water resource network. This enables accounting for transaction costs between individual buyer-seller pairs and abstractor-specific rules and behaviors using constraints. Trades are driven by economic demand curves that represent each abstractor's time-varying water demand. The purpose of the proposed model is to assess potential hydrologic and economic outcomes of water markets and aid policy makers in designing water market regulations. The model is applied to the Great Ouse River basin in Eastern England. The model assesses the potential weekly water trades and abstractions that could occur in a normal and a dry year. Four sectors (public water supply, energy, agriculture, and industrial) are included in the 94 active licensed water diversions. Each license's unique environmental restrictions are represented and weekly economic water demand curves are estimated. Rules encoded as constraints represent current water management realities and plausible stakeholder-informed water market behaviors. Results show buyers favor sellers who can supply large volumes to minimize transactions. The energy plant cooling and agricultural licenses, often restricted from obtaining water at times when it generates benefits, benefit most from trades. Assumptions and model limitations are discussed.

**Key Points:**

- Transaction tracking hydro-economic optimization models simulate water markets
- Proposed model formulation incorporates transaction costs and trading behavior
- Water markets benefit users with the most restricted water access

## 1 Introduction

Current and future regional water scarcity is stimulating efforts world wide to allow economically efficient reallocation of resources toward higher-value uses. Regulated market-assisted allocation of water is gaining support but the regulatory details of each regional market will need to reflect local water management, social and intuitional needs to receive widespread support. This creates a demand for the ability to understand and predict how water markets could perform under a range of hydrologic, institutional, and regulatory environments. Water markets' performance will depend on which transactions occur when, involving what quantity, and between which water right holders.

Modeling water markets realistically has remained elusive because of the challenge of representing how a multitude of actors interact amongst themselves, with institutions and with the spatially and temporally varying hydrological environment. Different approaches to modeling water resource systems with water trades exist in the literature. Hydro-economic models [*Harou et al.*, 2009] were the first to model combined spatially and temporally distributed hydrology and economic-driven allocation of water. Classical optimization-driven hydro-economic models have typically focused on the potential system-wide economic gains from water trading but have not represented individual level transaction detail of water markets and trading behavior. Griffin states that, in previous efforts to model water markets, “Too much is omitted to associate results with potential market results. The behaviors of individual agents (true market agents) are not represented, and the frictional transaction costs of market activity are neglected too” [*Griffin*, 2006, p. 356]. This has been the focus of new approaches that aim to represent more realistic “agent” behavior [*Berger et al.*, 2007; *Bonabeau*, 2002; *Giuliani and Castelletti*, 2013; *Yang et al.*, 2009; *Zhao et al.*, 2013]. “Agent” in this context refers to water users and in some cases a wider array of actors including institutional ones. Recent efforts in modeling innovative water market structures also include auction-based systems (smart markets), where each user trades with the auction manager [*Raffensperger and Cochrane*, 2010; *Schreinemachers and Berger*, 2011].

Recent contributions to water resource network modeling [*Cheng et al.*, 2009; *Erfani et al.*, 2013] allow accounting for the relationships between water sellers and buyers, i.e., track transactions in water resource networks. This allows optimization-based hydro-economic models, where movement of water is driven by hydrological inflows and time-changing demand curves, to get a step closer to simulating water markets because distinct user-defined transaction costs can be designed and introduced into the model for each pair of potential trading partners. Using a mathematical programming-based model formulation where each time step is an independent optimization model allows adding rules that help represent some degree of realistic agent (individual river abstractor) behavior. In this paper, water users have limited foresight of future river flows and make abstraction decisions for the following week based on the information available at the time. Hence, the optimization problem is solved sequentially on a weekly basis. The model takes the previous water availability, reservoir storage level, and catchment-specific conditions as input data prior to solving for each week. Afterward, water allocation is obtained only for the current week. This continues for all 52 weeks until the end of the year. The contribution of this paper is adding to an optimization-driven hydro-economic model (a) pair-wise transaction costs between all potential trading partners and (b) custom user rules in an effort to simulate potential water market behaviors.

Below section 2 presents the proposed approach and model formulation. Section 3 describes an application of the model to the Great Ouse river basin in Eastern England. This includes additional case-study-specific model constraints added to the basic model formulation. Results of simulations with and without the water market are presented in section 4. Section 5 discusses benefits and limitations of the proposed approach and is followed by conclusions.

## 2. Methods

The proposed model uses a node-arc multicommodity formulation following the transaction tracking method of *Erfani et al.* [2013]. The water resource system is represented by a network of nodes and links. Following classical hydro-economic optimization modeling practice, the model is driven by a single-objective optimization function to simulate water transfers that could occur in a market with perfect information. Perfect information refers to the fact that all agents know each other's value of water in each time step. To this are added rules, through use of constraints and penalties as explained below, to make water license-holder trading behaviors more realistic.

### 2.1. Proposed Approach

The proposed water market model is used to simulate short-term (spot market) trading behavior amongst individual water rights holders. Model predictions demonstrate intersectoral and intrasectoral reallocation of water and the resulting hydrological outcomes.

The model considers pair-wise trading by water rights holders who possess full information on marginal values of water of all other river abstractors in the system. A single-objective function means the model implements those trades which maximize regional economic benefits at each time step. The model assumes users with higher willingness to pay for water will buy from abstractors with lower marginal benefits if transaction costs do not sufficiently discourage it. The individual preferences of specific abstractors to trade or not trade with other users can be accounted for, if known, through detailed user-to-user transaction costs or rules imposed as constraints in the mathematical program.

A market with perfect information is a framework for approximating a “best-case” situation where all water rights holders would participate in the market using, for example, an online information and transaction system that allows viewing prices offered by others. In such a system trades would be preauthorized by an agency only after it could ensure transfers do not harm other abstractors or the environment.

The theory that market-based allocation of resources driven by self-interested individuals serves the common good of society dates back to Adam Smith and the concept of “invisible hand” [*Bennett*, 2005]. Agents act to maximize their own benefit, and in the process, scarce resources are allocated to the highest-value uses, maximizing the overall benefits to society as a result. For water markets to produce efficient allocation of resources, water rights need to be well defined and protected, and both positive and negative externalities need to be accounted for [*Freebairn*, 2005].

Allocative efficiency is achieved when marginal values of the resource in all uses are equal [*Bennett*, 2005]. This “equi-marginal” principle is expected to hold in a market characterized by perfect information and perfect competition. In market-based water allocation, however, marginal values are different across various types of use and locations of abstractors [*Cai*, 2008]. The reasons for this are institutional and hydrologic constraints on water trading [*Cai*, 2008; *Colby et al.*, 1993] such as the transaction costs and system market-constraining rules presented below.

### 2.2. Transaction Costs

*Griffin* [2006] posits that a model of water market would necessarily need to include transaction costs. Costs incurred, for example, to find trade partners and to study and execute transactions need to be reflected in the model. *McCann and Easter* [2004] set out a framework for analyzing costs associated with water exchange under different allocation mechanisms. For formal water markets, each stage of the trading process incurs costs. Costs that should be accounted for are associated with: (a) set up of the system, including legislation changes and design of the infrastructure, (b) pretrade costs of information gathering, (c) legal and professional contracting costs, and (d) costs of administering the system, including monitoring and resolving conflicts.

In the model presented in this paper, the costs are represented by a per-trade fixed charge and variable costs that increase with the volumetric size of the transaction, payable by the buyer. The fixed charge reflects cost recovery of cost types (a) and (d) by the agency running the system, whereas the variable costs reflect (b) and (c). The assumption is that variable costs increase roughly proportionally with the size of the transaction. We have modeled a situation where a regulator further taxes transactions following the consumptiveness of the buyer relative to that of the seller. That is, if the buyer's diversion produces lower return flows than that of the seller, the costs will be higher than if they were equal. This would account for some of the negative effects the transaction has on other users and the environment. At an aggregate level, after water is used, if a lower proportion of the volume abstracted is returned, less will be available for others to abstract. In other words, this regulation makes steps toward internalizing the negative externalities associated with the transaction. Section 3.4 details the transaction cost structure assumed in our case study.

### 2.3. Model Formulation

This section outlines the mathematical programming model formulation which allows representing pair-wise trading (see Appendix A for nomenclature).

#### 2.3.1. Objective Function

The benefit function quantifies total economic benefits generated by water abstractors from water use in each time step, expressed in monetary terms. Economic benefits from water abstraction for water users are represented as a function of allocation; benefit functions are obtained by integrating inverse water demand functions. All factors affecting users' water demand are exogenous and stay constant for our analysis.

The model includes a reservoir facility for water storage. The preference for specific storage levels throughout the year is enforced by penalizing deviation from the target level of reservoir. This can be considered an “agent” rule added to the model to ensure modeled behaviors are as realistic as possible.

The model is solved by maximizing an objective function variable: (1)
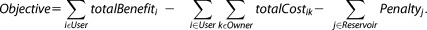


The *totalBenefit*_*i*_ generated by license holder *i* is a function of the total volume abstracted by user *i* in each time period: (2)
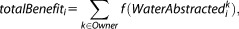


where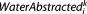 is the sum of water volumes abstracted using one's own license and purchased from other users.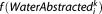 is a piece-wise linear quadratic benefit function derived from each user's inverse demand curve (see section 3.3).

The cost of trading incurred in each time period is the sum of fixed per-trade costs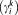 and per-Ml variable costs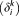 for each user *i* purchased from user *k*: (3)
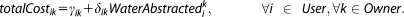


The last term in the objective function (1) is the absolute value of reservoir level deviation from its target multiplied by a penalty factor α (see section 3.2).

#### 2.3.2. Model Constraints

Generic constraints to represent water resource systems with trade transactions are listed below; specific constraints to reflect management rules and behaviors are described in the case-study section.

In the following equations, *i* belongs to the set of all users including agriculture, industry, water supply, and energy. Index *k* distinguishes between different types of water, which includes the river (licensed water in the river abstracted by the users) and the sellers' licenses (See Nomenclature in Appendix A for further details). In this formulation, index *k* is added to the flow decision variable to simultaneously describe physical water delivery network and water's origin and hence its ownership. Tagging ownership allows supplier-receiver transaction tracking.

##### 2.3.2.1 Mass Balance at Junction Nodes

Water balance at each junction node is ensured throughout the modeled river network. Water entering junction node i plus the inflows at the same node equals the water leaving node i: (4)
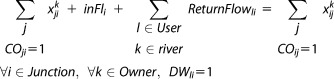


Since *inFl*_*i*_ is a river flow and initially belongs to no users, there is no need to index it by indicator *k*.

##### 2.3.2.2 Storage Balance

Storage balance states that the volume of water in reservoir in each time step equals the volume carried over from the previous time step, net of changes due to addition from river abstractions and water taken out of the reservoir for consumption. (5)
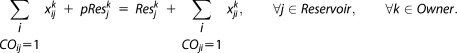


##### 2.3.2.3 Abstraction Balance

The user node consumes 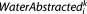, including water from its own license and potentially from buying from other water right holders, and sells the rest of its licensed water volumes to others (*Trade*). (6)
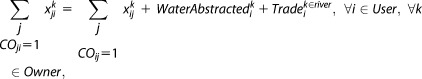
where (7)
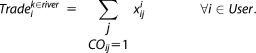


Variable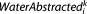 represents the sum of water bought and abstracted using one's own license. To track the transaction from the original license holder to the recipient, the positive variable *Trade* is added to the node mass balance equation of the original license holder. Therefore,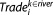 is the volume of water transferred from the user i down the river to the recipient(s).

##### 2.3.2.4 Return Flows

Some water uses are not fully consumptive, and water leaves the user node and is returned to the river for abstraction by users downstream (*Return flow*). (8)
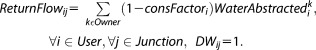


Once return flow is calculated, it is balanced using the junction nodes set of equations (4). Return flows are assumed to come back to the river without ownership and hence there is no need to flag it using index *k*.

##### 2.3.2.5 Mass Balance at Discharge Zone

Excess water is discharged at the final node (sink) downstream of the river section. (9)
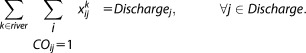


##### 2.3.2.6 License Constraints

The volume of water allocated to each license holder can either be abstracted for local consumption or sold to other users. In other words, the sum of the water volumes abstracted using one's own license and sold cannot exceed total licensed amount per time step. (10)
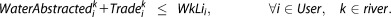


Annual cumulative consumption is tracked and water right holders cannot exceed their annual volumetric allocation.

A sale can be made only to users located downstream of the seller, along the same river or tributary, or from tributary to the main river. This is done to minimize trade-induced third party license derogation, i.e., when a sale upstream prevents water users located between the buyer and seller to achieve their intended diversion.

## 3. Great Ouse River Case Study

**Figure 1:**
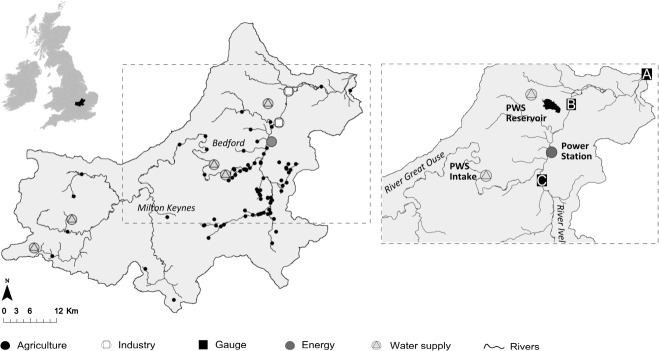
Approximate active abstractor locations and main flow observation gauges represented by points A (last gauge in the basin, sink), B (Offord gauge), and C (gauge, defining abstraction restrictions for the power station license).

This section introduces the case-study river basin and describes further constraint equations added to the proposed model to reflect local water management. The model was applied to the 3000 km^2^ Upper Ouse and Bedford Ouse river basin of Eastern England (Figure 1). Annual rainfall averages range from 670 mm (west) to 540 mm (eastern catchment) [*Environment Agency*, 2005].

### 3.1. Water Use

Data on 205 surface water abstraction licenses (water rights for diversion and consumptive use) and monthly volumes abstracted by license holders over 2006–2011 were obtained from the Environment Agency (EA). There were no abstractions over that period for 111 licenses which are assumed unused. Although the EA allows trading of unused licenses in some basins [*Environment Agency*, 2011] because the Great Ouse is overlicensed and overabstracted we assumed dormant licenses cannot be traded in our model runs. The remaining 94 river water abstraction licenses were represented individually in the model. Licenses were divided into four categories: Agriculture, Public and Private Water Supply, Industry, and Energy. Table1 provides summary statistics on the active licenses and Figure 1 shows approximate abstraction locations.

**Table 1:** Active Abstraction Licenses in the Great Ouse Basin

Sector	Percentage of the Total Number of Active Licenses Held by Sector	Percentage of the Total Yearly Volume Licensed for Abstraction Held by Sector
Agriculture	91.5	1.7
Public and private water supply	5.3	93.7
Industry	2.1	<1
Energy	1.1	4.5

The model includes an energy sector abstractor: a gas power station using water for cooling. Two surface water abstraction licenses are held by the regional public water supply (PWS) company. One is used to fill the PWS Reservoir (Figure 1) and another is a direct river intake upstream of the reservoir (Figure 1).

### 3.2. Water Allocation

This section describes current and potential future water management arrangements in the catchment and in England and Wales generally and how they are represented in the optimization model constraint set.

#### 3.2.1. Water Licensing System

Diversions (abstractions) of water from surface water and groundwater sources in England and Wales are regulated by the Environment Agency (EA). Water allocation is managed through a licensing system whereby an abstractor obtains a license specifying the conditions under which water abstraction is authorized. These conditions include the volume of water permitted for abstraction, water use description, location of abstraction, and special conditions under which abstraction is to be reduced or suspended.

The two major cases under which abstraction is prevented are Hands-off (environmental) flows and rules defined in Section 57 of the Water Resources Act, 1991 (Section 57). Hands-off Flow (HoF) rules are conditions on licenses that specify the minimum flow in the river below which the affected licenses must reduce or stop their abstractions [*Environment Agency*, 2012c]. The purpose of HoF rules is to ensure water availability for priority uses and to protect the environment during drought. Section 57 rules describe emergency provisions for managing spray irrigation licenses in case of drought. These provisions limit or prohibit abstractions for the purposes of spray irrigation to ensure public water supply needs are met [*Environment Agency*, 2012a].

#### 3.2.2. Modeling License Restrictions: Hands-Off Flows and Section 57

If flow at a flow observation gauge is less than the minimum flow set by a HoF rule specified in the license, the abstractor is not allowed to divert water from the river or sell any part of the license. (11)
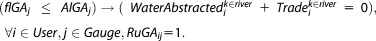


There are two major HoF rule types applied in the basin. The first is the rule termed the “Offord Clause” that refers to the flow passing through the gauge located near Offord (Point B in Figure 1). It protects the PWS Reservoir license held by the local public water supply company to ensure other licenses do not derogate it. The second type is based on local flow conditions. These HoF conditions set minimum flow requirements for 11 gauges distributed throughout the basin. Twenty eight active modeled licenses are affected by HoF conditions below which licenses are temporarily suspended.

Under Section 57 of the 1991 Water Resources Act agricultural abstraction can be reduced by the EA under dry conditions when PWS is under threat. In the model, this is represented by a 50% reduction in weekly abstraction by spray irrigators when river flow is lower than the flow which is exceeded on average 95% of the time (Q95). Agricultural users who face this temporary decrease in their licensed volumes are located upstream of the corresponding gauging station recording low flow. (12)
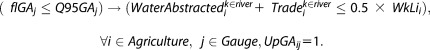


The above if-then conditions (equations (11) and (12)) are checked offline prior to solving for each time step of the model.

#### 3.2.3. Public Water Supply Rules

The PWS reservoir operation is modeled using a set of rules. The first are volumetric capacity constraints (in millions of liters): (13)
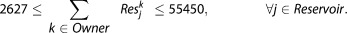


Withdrawals from the reservoir are only allowed if the reservoir storage is above the minimum volume.

**Figure 2:**
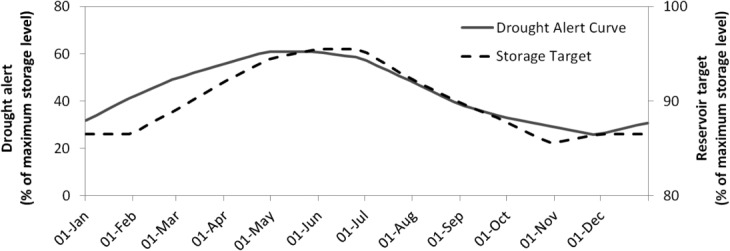
PWS storage target and the Drought Alert Curve.

The reservoir has seasonal storage targets (Figure 2). The model represents PWS's preference for these storage levels by penalizing storage target deviations in the objective function. Penalty factor *α* is calibrated using the historical profile and operational details for the PWS Reservoir. (14)
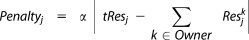


**Figure 3:**
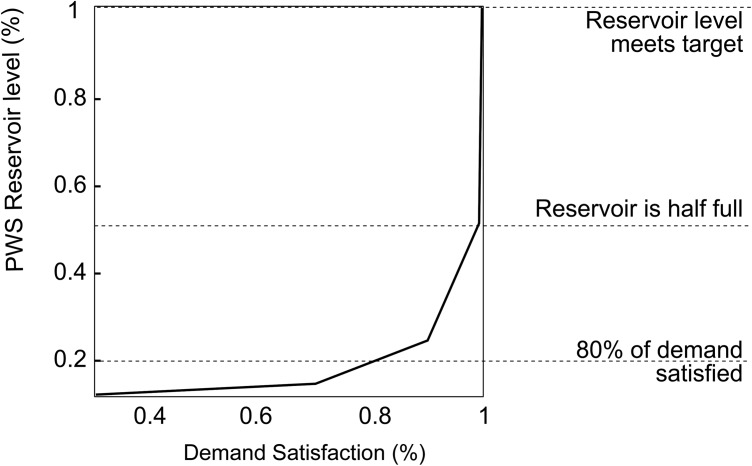
Public Water Supply company hedging rule.

In a drought public water supply companies introduce water saving initiatives, such as hosepipe bans, to ensure basic water needs can be met. To reflect this, the model uses a reservoir hedging constraint in addition to the target storage deviation penalties. The larger the deviation, the smaller the withdrawals from the reservoir become. The constraint introduced to reflect this hedging rule is: (15)
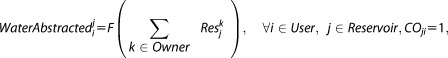


where *F(.)* is the function shown in Figure 3 which represents the relationship between the reservoir level, as a percentage of the target, and the proportion of the demand that is satisfied.

The two PWS licenses are connected by a rule on trading. When the PWS Reservoir storage volume is low, the PWS intake license manager is not expected to sell any water. In the model, if the reservoir level is below the drought alert curve shown in Figure 2, any further water sales are prohibited, until the level recovers. This condition is checked offline prior to solving for each time step of the model. It is noted that once water is abstracted into the reservoir it is stored in PWS Reservoir and is only taken out by the public water supply company for consumption.

#### 3.2.4. Water Trading

Currently license trading is permitted in England and Wales but rarely carried out. In 2003–2008, the Environment Agency registered 48 water license trades in England and Wales [*HRWallingford*, 2012], out of which 31 trades were carried out in the East Anglia region where the Great Ouse River is located. An application for a permanent license takes 3–4 months to be considered, and a temporary license—28 days [*Environment Agency*, 2008], although, in practice, many water trade transactions take around 6 months. This means short-term spot-market trading is not possible now, but it is being considered by the EA which is investigating mechanisms to support water trading, including preapproved trades and a modern licensing system [*Environment Agency and Ofwat*, 2012]. At present, the entire water license or a part of the licensed volume can be traded between a buyer and seller, for a set period of time, or indefinitely. If the whole license is sold, the seller gives up his license, and the buyer applies for a new license, or amends the license currently held for a fixed charge of £135 [*Environment Agency*, 2011]. To carry out a permanent trade, the buyer applies for a permanent license, and for a temporary trade, a temporary license. When the buyer's temporary license expires, the seller's license is restored to its original volumetric limit.

Discussions on early model results with basin stakeholders [*HRWallingford*, 2012; *Lumbroso et al.*, 2014] revealed a concern that some abstractors, particularly farmers, were “trading themselves out of business” (e.g., selling most of their annual licensed volume before the irrigation season). Without requiring intertemporal optimization, or complex submodels for each abstractor type, a limit on volumes sold by agricultural users was set. At each time period *t*, (16)
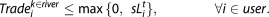


This selling limit (*sL*) applies until the farmer abstracts a proportion *c_i_* of the expected water need (*EWN_i_*), which is based on their historical yearly water use. For each user *i*, (17)
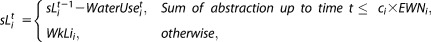
where *WaterUse* is the sum of water diverted and sold, *c_i_* is a value ranging from 0 to 1, *WkLi* is the normal weekly license limit, and (18)
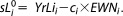


This constraint sets aside a portion of the yearly license for own use and can ensure abstractors do not sell excessively forcing them to purchase water later in the year. *c_i_* can be considered a “trade reluctance” coefficient representing the degree to which farmers prefer to save water for their own later use, and can be set for each abstractor. This coefficient allows the analyst to consider a range of market participation behaviors (0 if the abstractor trades whenever it is economically beneficial regardless of future needs; 1 if abstractors are conservative and first satisfy their irrigation demands).

The water market is simulated using the formulation proposed in sections 2 and 3.2. In our proof-of-concept case study, we lacked a detailed study on farmer attitudes toward trading water so *c_i_* was set to 0.5 for all agricultural abstractors. Additionally a no-trading base-case scenario is modeled using a priority-based allocation [e.g., *Draper et al.*, 2004] to assess the impact of trading. In that formulation abstractors are prioritized from upstream-to-downstream while considering HoFs and Section 57 reductions to emulate current water management practices.

### 3.3. Estimating Economic Water Demands

The point expansion method was used to estimate linear demand functions for the water supply, agriculture and industry sectors [*James and Lee*, 1971; *Griffin*, 2006]. For energy plant cooling, the marginal water value is assumed constant and the resultant demand curve is a horizontal line. The total benefit functions are calculated by integrating the demand functions, and are, therefore, quadratic for all sectors except for energy, for which they are straight upward-sloping lines.

A demand function was estimated each week of the year for each abstractor. The original point of expansion is based on average actual weekly volume abstracted and marginal value of water. A function is then derived using literature estimates of price elasticity of demand. Currently observed volumetric charges for water abstractions in England and Wales are set to cover administrative costs and are not set by a market and thus do not reflect the economic value of water. Therefore, in this application, water values were adopted from past research. Table2 shows marginal values and price elasticities of water demands used in the study.

**Table 2:** Marginal Values and Price Elasticities of Demand for Water Used to Build Demand Curves for Each Water Diverter in the Case Study

Sector	Marginal Value in £/m^3^ (Elasticity of Demand)	Sources
Water supply	1.3 (−0.14)	Value derived using Gibbons' Gross Willingness to Pay formula, using £1.45/m^3^ (Anglian Water charges to households for metered water [*Anglian Water*, 2012]) [*Gibbons*, 1986; *Moran and Dann*, 2008], elasticity of demand—*UKWIR* [2003]
Agriculture	Sep to Mar 0.017	Values derived from *Knox et al.* [2000], using main-crop potato value and distribution of benefits across growing season. Low winter value—based on *Morris et al.* [2003]. Elasticity of demand—*Scheierling et al.* [2006]
	Apr to May 0.6	
	Jun—0.53	
	Jul—0.28	
	Aug—0.1 (−0.16)	
Industry	1.86 (−0.16)	Value derived from average value for golf industry in Spain [*Diaz et al.*, 2007; *Scheierling et al.*, 2006]
Energy	13 (N/A)	Value derived using residual imputation method based on wholesale price of energy, water requirements for power generation using CCGT technology and costs of energy production. The value compares well with other estimates [*ACILTasman*, 2007; *Maulbetsch and DiFilippo*, 2006]

The weekly demand curves for water users were estimated based on the same marginal value across all users within the same sector. The volume abstracted by the user determines the total benefit from water use. The demand function pivots around the *y* axis depending on the volume of water consumed.

There are no ecological demands such as those represented by *Yang et al.* [2009] and environmental flows are not assigned with economic values. Instead, environmental requirements as enforced by the Environment Agency are included as constraints (HoF conditions and Section 57 provisions).

### 3.4. Setting Transaction Costs

The formulation allows introducing customized transaction costs for each individual trade (i.e., each week, the transaction cost between each individual pair of buyer and seller can be set) to represent market friction. Under the current system of water right transfers in England and Wales, the buyer incurs administrative costs set by the Environment Agency and the seller is not charged.

The transaction costs in our application include a one-off set price for entering into a trade and a volumetric charge that differs according to the consumption factor of the buyer relative to that of the seller. The fixed cost was set at £13.5, a tenth of the current charge, and represents an administration charge. The variable cost used in this study combine costs of pretrade information gathering, contracting costs and an administrative charge aimed to reduce consumptiveness. Water trade transaction costs estimated by *Hearne and Easter* [1997] in Chile are an equivalent of 69$/ML (million liters). *Archibald and Renwick* [1998] differentiate between explicit administrative costs and policy-induced costs associated with measures to prevent negative externalities on other users and the environment in California's water market. Administrative costs are 14–41$/ML and policy-induced costs are 44–152$/ML. In policy-induced transaction costs *Colby* [1990] includes charges incurred in the process of obtaining the authority's permission of changing the location and purpose of abstraction, which includes legal fees and costs of engineering and hydrologic studies. Estimates of these transaction costs in the United States are on average 73$/ML [*Colby*, 1989].

The water transfer system in the proposed model is based on weekly trades that would be preapproved by the environmental regulator. Taking this into account, transaction costs are expected to be lower than those estimated in the above studies, where each trade has to be considered on a case-by-case basis, at the time of application.

The variable cost element of the transaction cost takes into account the effect the transfer has on the environment by incorporating the differences in consumption level of the buyer and the seller, and is proportional to the volume traded. We model the case where the environmental regulator would levy a charge to discourage trading toward more consumptive uses. This variable charge was calculated as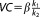 where *k*_1_ and *k*_2_ are the consumption factors (fraction of water evaporated relative to diverted) of the buyer and the seller, respectively. Constant *β* is a proxy for the costs the buyer incurs in negotiating a transfer with the seller and was set to £12.5 (a tenth of the hourly charge currently levied by the EA for advice on trading a water license [*Environment Agency*, 2012b]). These variable costs penalize leases from the users with lower to the users with higher consumption factors. Table3 shows the variable costs associated with transactions between sectors used in the study.

**Table 3:** Variable Transaction Costs, £/ML

Seller Buyer	Agriculture	Water Supply	Industry	Energy
Agriculture	12.5	12.5	20.8	25
Water supply	12.5	12.5	20.8	25
Industry	7.5	7.5	12.5	15
Energy	6.3	6.3	10.4	12.5

### 3.5. Water Price Determination

The price of water in each trade was estimated using the following formula: (19)
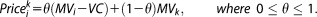


The unit price of a water trade should be between the willingness to pay for water of the buyer (minus variable transaction costs) (*MV*_*i*_ – *VC*) and the seller (*MV*_*k*_). In this study θ is taken as 0.5; the price is the average of the willingness to pay of the two trading abstractors (once the buyer's transaction costs have been removed).

### 3.6. Determination of Net Benefits With Transfers of Water Payments

Water trade payments are determined as a product of the price and the quantity traded. Net benefit from water use considering water market transactions is calculated as follows: (20)
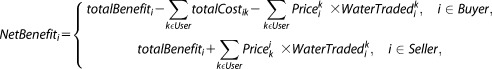


where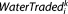 is the volume of water bought by user *i* from user *k* and 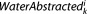 is the volume sold by user *i* to user *k*.

### 3.7. Hydrological Flows

**Figure 4:**
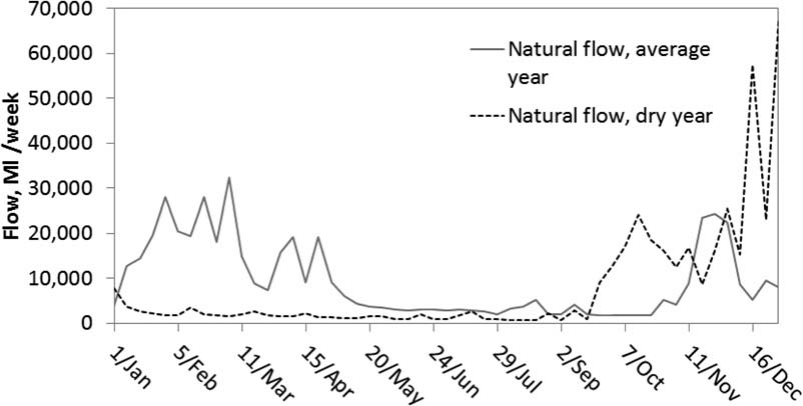
River flow at the last gauge in the basin (marked A in Figure 1).

The model is run for a historical dry and normal year. Figure 4 shows natural flow levels (without considering human river diversions) at the basin exit, (marked A in Figure 1) for both years. The dry year is characterized by very low flow until mid-September; the average year flows are higher overall although the summer is still relatively dry.

## 4. Results

In this section, we apply the proposed formulation to model trading (i.e., weekly pair-wise water rights leasing of abstraction licenses) in the Great Ouse water resource system for a normal and a dry year. A scenario without trading and using current allocation procedures helps assess how the modeled trading affects water use and river flows.

### 4.1. Trading Results

**Figure 5:**
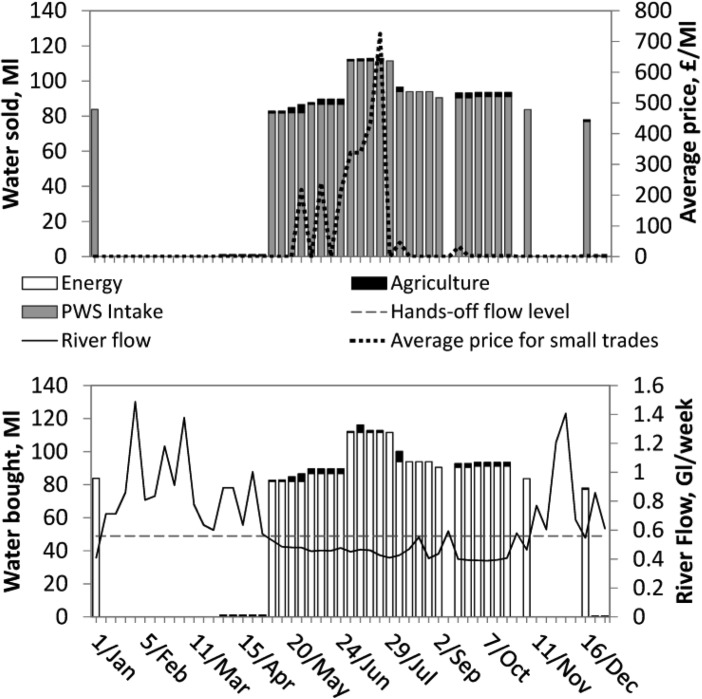
Water volumes (top) sold and (bottom) bought in Millions of liters/week by sector in the normal year. Top plot displays average weekly prices estimated for trades between water users excluding higher priced large trades between PWS intake and the power station. River flow in billions of liters (Gigaliters, GL) per week displayed (bottom plot) is at gauge C (see Figure 1).

**Figure 6:**
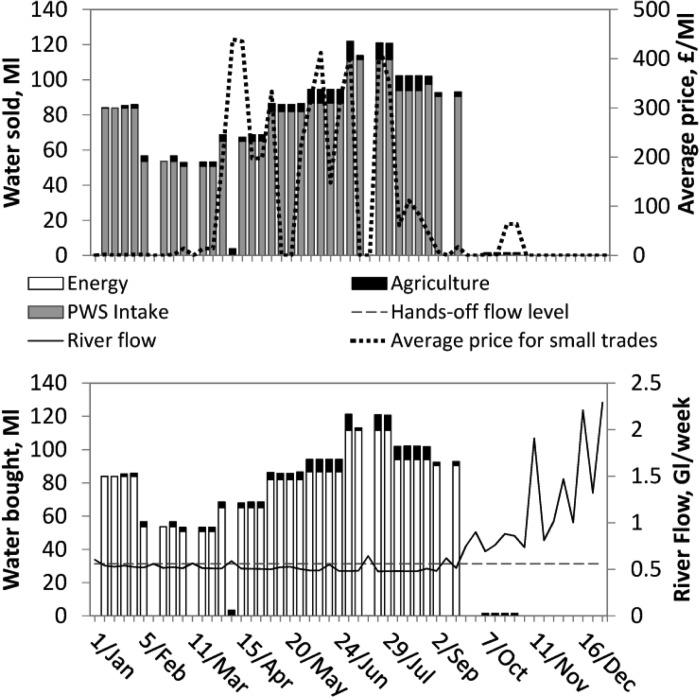
Water volumes (top) sold and (bottom) bought in Millions of liters/week by sector in the dry year. Top plot displays average weekly prices estimated for trades between water users excluding higher priced large trades between PWS intake and the power station. River flow in billions of liters (Gigaliters, GL) per week displayed (bottom plot) is at gauge C (see Figure 1).

Figures 5 and 6 show bought and sold water volumes aggregated by sector for the normal and dry year. These figures only include sectors transferring over 1 ML/week. In both years, the largest transfers of water allocations by volume are made from the public water supply company to the energy sector, comprising 99% and 94% of the total volume transferred in the normal and dry year, respectively. The power station's license is affected by Hands-off Flow conditions defined with reference to gauge C (Figure 1). When the river flow at gauge C (shown in the Figures 5 and 6, bottom) is below Hands-off flow, the power station's license is temporarily suspended, and this user satisfies its water demand by buying from the public water supply company.

Even though agricultural marginal water values are the lowest of the four sectors, the results do not show significant reallocation of water from agriculture to higher-value uses. Over 90% of water sold by agricultural users was transferred to other agricultural users. This is a result of the transaction costs assumptions posed. Each trade attracts a fixed charge that discourages large abstractors such as power stations from buying multiple small allocations from farmers.

River flows affect trading frequency, with the drier portions of the years sustaining most trade. Figure 6 shows that transfers stop in mid-September of the dry year. The reason for this is higher availability of water from this point onward which means license constraints no longer prevent water diversion. This also occurs in Figure 5 with less water traded in the wetter winter and spring seasons.

The proposed pair-wise trading formulation implies each individual water trade has its own price, “negotiated” between the two water users. Figures 5 and 6 (top) show weekly prices, averaged across all trades excluding the power station's purchases from PWS intake. The estimated price for trades between the PWS intake and the power station is £6500/ML. This is because the demand function for the power station was taken as a horizontal line, and the PWS intake's demand is fully satisfied through the year, and the willingness to pay is, therefore, 0. The average prices for trades between all other users range between almost 0 to £725 in the normal year and £440 in the dry year.

**Figure 7:**
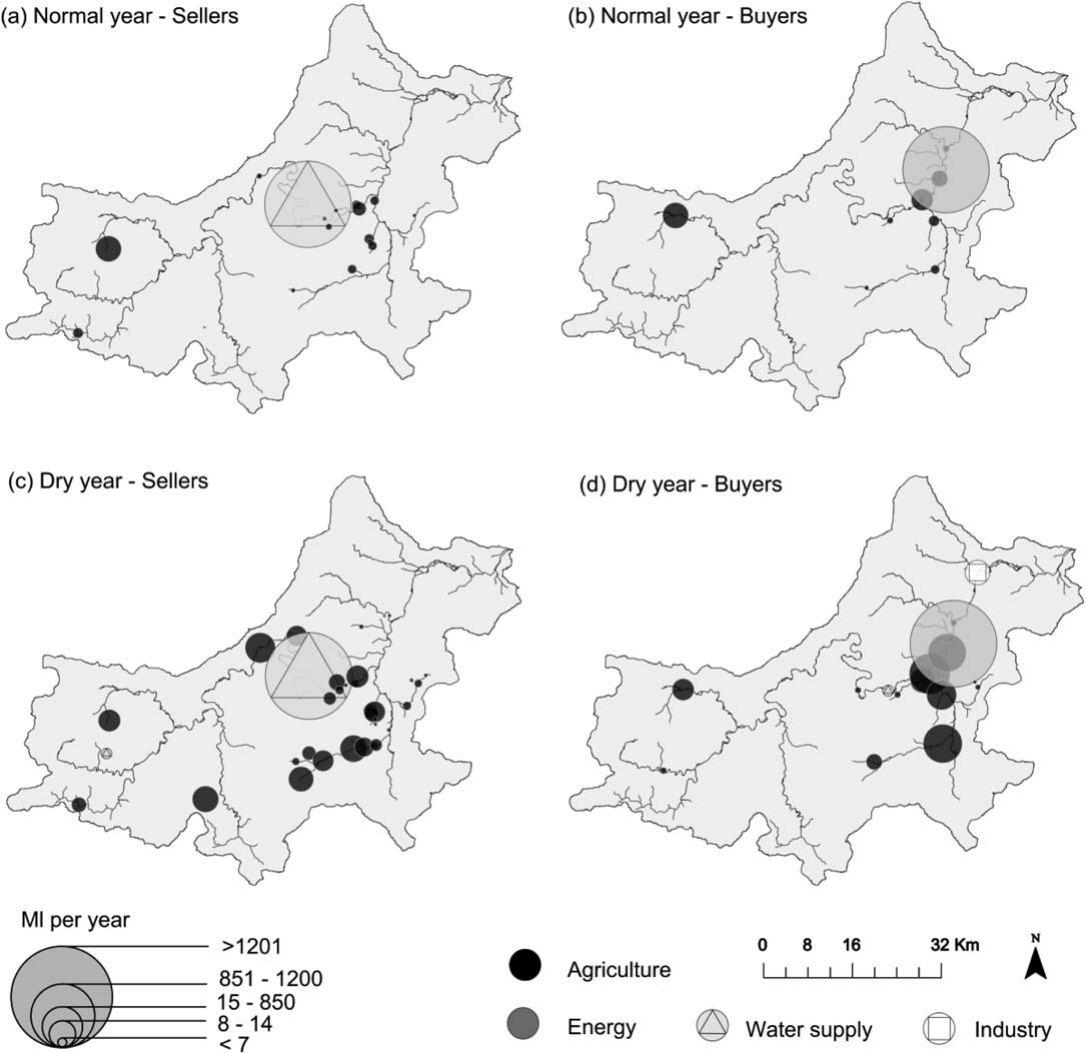
Locations of buyers and sellers of the normal and the dry years' model applications.

Figure 7 shows locations of sellers and buyers with circle size proportional to transferred volume. Most buyers are downstream of Bedford, and along the River Ivel (see Figure 1). This part of the basin is characterized by high density of agricultural users and sensitivity of the river to abstractions. There are 13 water dependent “Sites of Special Scientific Interest” (SSSIs), such as river-fed lakes, moors, and meadows. This is reflected in strict HoF restrictions on the licenses in this section of the basin. Under the current licensing regime, tributaries of the river Ivel are overlicensed, and the region's groundwater resources are overabstracted in a normal hydrological year [*Environment Agency*, 2005]. This further increases the abstractors' reliance on surface water resources. In the modeled dry year, this produces high volumes of transfers to abstractors most affected by scarcity.

As explained in section 3.3, the demand curves for agricultural water users were estimated based on the same marginal value. Hence, when a Section 57 or HoF rule is activated, the affected users have a higher value of water. Trading between farmers is driven by the market's tendency to bring marginal values as close as it can.

Only one agricultural abstractor reaches its yearly abstraction limit in the dry year scenario. If the selling limit rule (section 3.2.4) is omitted, this results in a 0.24 ML/yr (0.2%) volumetric increase in water sold by the farmers. This outcome is determined by the existence of a large license not constricted by HoF conditions (the PWS license), which sells to the power station. If the PWS license did not sell, the next-best solution is for the power station to buy from irrigators. In that case the selling limit would have a larger effect on farmer behavior and the reluctance coefficient (section 3.2.4) would affect the trading results more.

### 4.2. Effect of Changes in Fixed Transaction Costs on the Benefits From Water Use

The fixed transaction costs of £5, £10, and £20 were tested to observe their impact on trading. Table 4 shows that the number of trades reduces progressively as the fixed charge increases. The size of the fixed charge influences whether abstractors engage in trading. With higher charge, smaller users trade less and large water users purchase water from a smaller number of sellers.

**Table 4:** The Effect of Changes in Fixed Transaction Costs on the Number of Trades

	Fixed Charge	£5	£10	£13.5	£20
Number of trades	Normal year	186	178	147	137
	Dry year	332	315	299	284

There is no effect of changes in fixed transaction costs on the power station's and PWS intake's water use and gross benefits from water use. Water use does not change because the magnitude of the transaction cost is low in comparison with the high benefit from water use at the power station (transaction costs as postulated are less than 0.03% of the gross benefit from water use), and PWS intake's water demand is fully satisfied in both the dry and the normal year simulations without the need for water purchases. The effect on other users involves a slight shift in distribution; as some users decrease water purchases and water abstractions it enables downstream users to increase abstractions. With higher fixed transaction costs, small agricultural users reduce their abstractions and water purchases allowing the PWS Reservoir to increase abstractions and the industrial sector to purchase higher volumes of water. Table5 shows the effect of the increase in fixed transaction cost from £5 to £20 on benefits from water use, volumes abstracted and volumes traded.

**Table 5:** The Effect of Changes in Fixed Transaction Costs From £5 to £20 on Water Abstractions (Ml), Volumes Bought (Ml), and Gross Benefits From Water Use (£; % Increase/Decrease Due to Increased Fixed Transaction Cost)

Sector		Dry Year	Normal Year
Agriculture	volume abstracted (Ml)	−43 (−5%)	−26 (−3%)
	volume bought (Ml)	−1.8 (−1%)	−9.5 (−13.5%)
	benefit from water use	−£5000 (−1%)	−£2600 (−0.5%)
Public and public water supply	reservoir intake (Ml)	28 (<0.01%)	1000 (<0.01%)
	abstractions from reservoir (Ml)	1.7 (<0.01%)	22 (<0.01%)
	sector benefit from water use	£3400 (<0.01%)	£49,800 (<0.01%)
Industry	volume abstracted (Ml)	0.02 (0.2%)	
	volume bought (Ml)	0.02 (0.2%)	
	benefit from water use	£12.5 (<0.01%)	
*Overall*	*benefit from water use*	*−£1587.5*	*£47,200*

Agricultural users' trading is more sensitive to fixed cost changes in the normal year than in the dry year. The volumes bought in the dry year reduce by 1% and in the normal year by 13.5% under higher transaction costs. Because of the higher level of water scarcity in the dry year, the marginal values of water in the dry year are higher and the fixed cost change has less effect on trading. Also, the agricultural sector's reduction in benefits from water use due to increased fixed transaction cost is higher in the dry year than in the normal year (£5000 reduction in the dry year, £2600 in the normal year). The effect on the volume abstracted by agricultural users is larger than on the volumes bought. As smaller farmers stop purchasing water, larger agricultural users remain in the market, purchasing larger volumes of water, decreasing the ability of nearby farmers to access water.

Less abstraction by agricultural users increases the river flow past the PWS Reservoir. PWS Reservoir increases river abstraction and the public water supply company increases reservoir abstraction because storage volumes are closer to the target levels. This leads to a counterintuitive overall increase in economic benefits for the public water supply sector in the case of higher transaction costs. The overall impact of increased fixed transaction cost on economic benefits from water use on all sectors depends on the exact mix of demands and their relative locations. In our case-study system, economic benefits decreased by around £1600 in the dry year but increased by £47,200 in the normal year; a breakdown by sector is provided in Table5.

### 4.3. Application With No Trade

**Figure 8:**
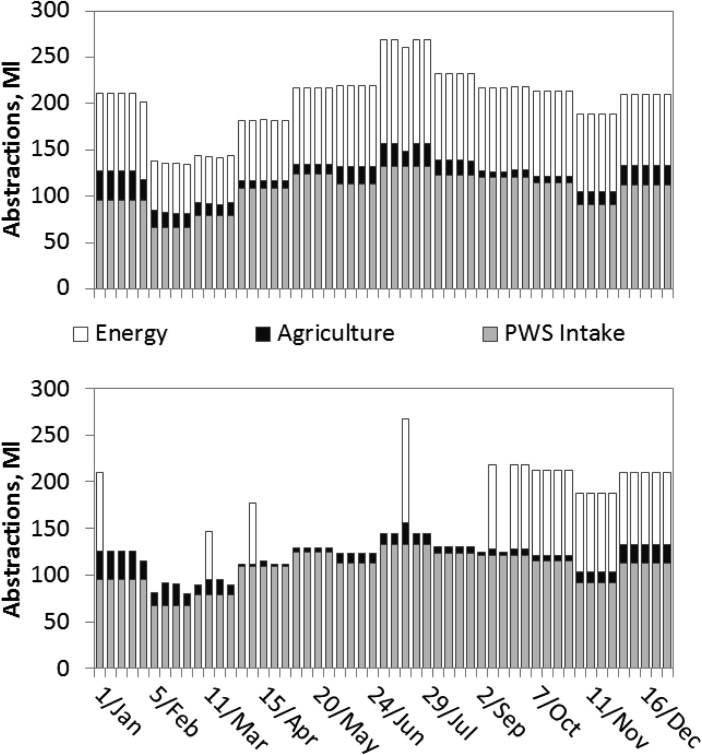
Total abstractions, by sector in dry year with (top) trading and (bottom) no trading.

To investigate how water consumption by each river abstractor is influenced by trading we ran the model eliminating the possibility of trading. The problem is converted to a simple upstream-to-downstream allocation scheme using historical demands. Figure 8 compares abstractions by the main sectors (abstracting more than 1 ML/week) in the dry year with trading (top graph) and without (bottom graph).

Without the water market the power station is unable to abstract in most dry year weeks. When the two scenarios are compared, the difference in volumes abstracted by the power station is attributable to purchases from the PWS license. The agricultural sector also has almost 20% lower annual abstraction volumes when trading is excluded. This is particularly true during the summer growing season. In the dry year, the spot market facilitates movement of water between abstractors and the resultant abstraction profile changes substantially.

Public Water Supply abstractions are not affected by trading as much as other sectors. PWS abstractions are the same regardless of the availability of weekly trading because their high priority water rights (no HoFs) mean they are never required to seek alternative supplies. The PWS abstractor is also a major seller in the trading scenario.

**Figure 9:**
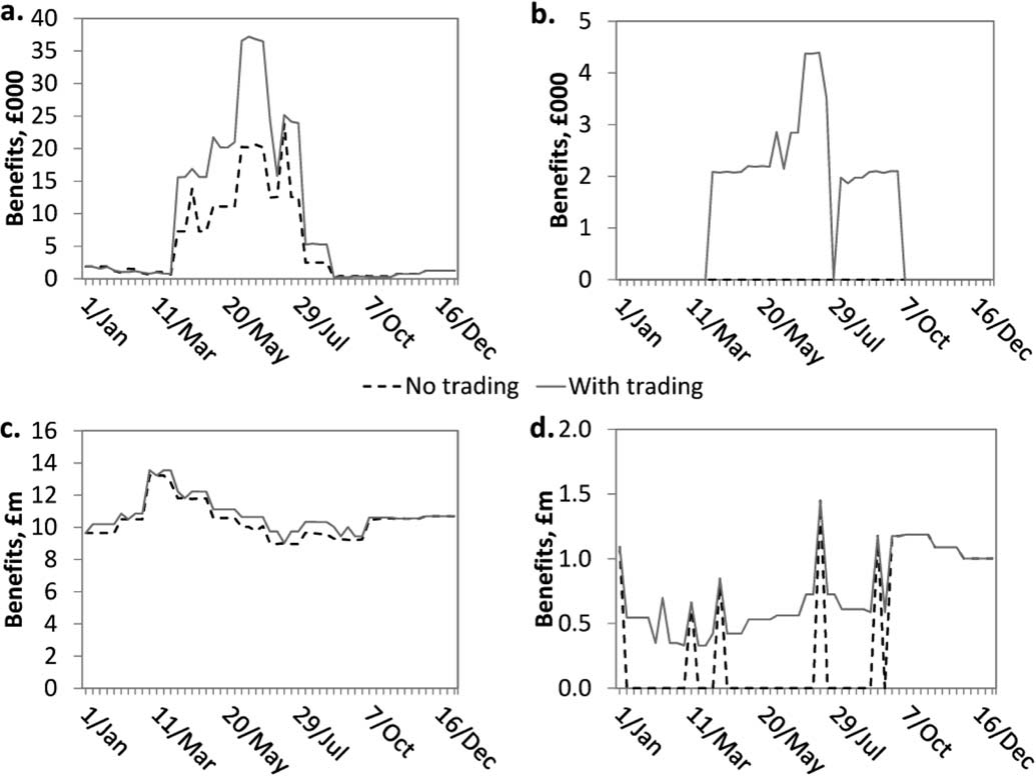
Comparison of economic benefits from water use net of transaction costs and payments for traded water aggregated by sector with and without trading in a dry year: (a) Agriculture; (b) Industry; (c) Water supply; and (d) Energy. Note differences in *y* axis scales; the agriculture and energy sectors benefit most from trading in a drought year.

Figure 9 shows the benefits net of transaction costs and payments for traded water across the four sectors (see section 3.6 for method of determination of net benefits with transfers of water payments). Each of the four sectors' net benefits are increased when short-term trading is permitted. For each week when the power station purchases water allocations from PWS intake, half of the benefit from water use by the Power station is transferred to the PWS intake in the form of payments for water. Hence, the water supply industry's net benefit increases when trading is introduced.

## 5. Discussion

Results of the case study showed that buyers tend to favor sellers who can supply large volumes and minimize the number of transactions. The abstractors likely to benefit the most from trading are those that currently have newer more restricted licenses that prevent them from obtaining water at times when it generates benefits (mostly energy cooling and agricultural licenses in our case). Although the reluctance to trade coefficient and the selling limit rule did not have a large impact in the case study due to the large power station's demand being satisfied by purchases from PWS, the formulation allows representing a range of trading attitudes without having to know the exact reason behind them.

The Great River Ouse basin in the case study has two users dominating the market, one as a seller (PWS intake), and one as a buyer (power station), accounting for 99% and 94% of volumes transferred in the normal and the dry year simulations, respectively. The presence of two large water abstracting traders in the system affects water prices paid for water by other users. If the power station did not participate in trading, PWS intake would sell to smaller users, decreasing the prices paid for water by agricultural, industrial and private water supply users. Conversely, if the public water supply company did not participate in the market, the power station would buy water from smaller users and its high willingness to pay would mean that some small buyers would be priced out of the market.

Table 6 summarizes the total economic gains net of transaction costs and payments for water trades from the surface water spot market by sector in the dry and normal modeled years. The limitations of the proof-of-concept case-study application discussed below suggest what issues might be addressed to increase precision of results.

**Table 6:** Increase in Economic Benefits From Water Use Net of Transaction Costs and Payments for Traded Water Due to Trading

Sector	Increase in Benefits (£Million/yr), Normal Year	Increase in Benefits (%), Normal Year	Increase in Benefits (£Million/yr), Dry Year	Increase in Benefits (%), Dry Year
Public and private water supply	16	3	18	3.2
Agriculture	0.04	8	0.2	67
Energy	16	68	17	81
Industry	0.07	N/a	0.06	N/a

The suitability of single-objective optimization models for simulating river basin water allocation has been questioned in recent research because they represent a system-wide optimal solution which does not realistically represent individual actions [*Yang et al.*, 2009]. Each water user maximizes their own benefit, and the notion that users would work cooperatively to maximize the total welfare of the region is not expected in real-world systems. Aggregate optimization and individual optimization methods have been compared by *Kuhn and Britz* [2012]. The allocations of water simulated by the two methods differ for nonmarket setups. For tradable water right simulations, however, the water allocations results are the same. *Britz et al.* [2013] state that the aggregate optimization method is “appropriate as long as interactions between agents and competition for resources can be interpreted in a competitive market paradigm.” Water markets eliminate allocative inefficiencies [*Kuhn and Britz*, 2012], however, in real-world water markets, transaction costs prevent the optimal allocation from being achieved [*Garrick and Aylward*, 2012; *Garrick et al.*, 2013]. Our model simulates water transactions that reduce allocative inefficiencies, subject to institutional and physical constraints and possible transaction costs. *Takayama and Judge* [1971] show that “both a welfare maximum optimum and a Pareto optimal point satisfy market equilibrium.” Single-objective optimization with transaction costs, therefore, can be considered an appropriate method to assess market-based allocations of water.

The Great Ouse river basin has 128 groundwater licenses. Groundwater flow, its interaction with surface water abstraction, conjunctive use with surface water and groundwater trading were not modeled in this exercise; their inclusion could be of interest but is beyond our scope. Most groundwater licenses in the basin are used fully and new licenses are unlikely to be issued so it is likely that a regional water market would not heavily change how groundwater is used.

The model's results are driven by (a) the economic demand curves used to represent each abstractor's water demand; (b) the transaction costs assumed between buyers and sellers; and (c) abstractor water management practices represented by constraints. Transaction costs were set to estimates of fixed and variable costs users could incur if a system of preapproved short-term trading was established in England and Wales. The modeling framework allows the analyst to represent more sophisticated abstractor preferences by tailoring transaction costs between any trade pair and varying these in each time step. For example, negotiating multiple trades with a large number of small users could take a substantial amount of time for a large industrial user. The transaction costs in the current formulation make steps toward representing this time spent on negotiation in monetary terms, by fixed cost. Also, the rules entered as constraints allow flexibility to represent any number of criteria that overrun economic considerations as represented by the demand curves and transaction costs. As with all modeling, and particularly those representing human behaviors and decisions, the model is only as good as the data and assumptions built into it. The case study would benefit from a refinement of the demand curves, abstractor rules, and transaction costs used to represent abstractor preferences. The question in models such as these is how correct must these be to enable the model to produce valuable insights about how the system would perform under different policies (e.g., licensing regimes and trading rules). In this case study, we have opted for simple methods (linear demand curves, transaction costs that only vary by abstractor type) to ease result interpretation.

This paper brings up the issue of whether single-objective function (“centralized” optimization) is appropriate for modeling river basin water management, or if newer decentralized methods are more appropriate. In recent efforts to model water resource allocations, agent behavior has been represented within a decentralized optimization-based framework [*Yang et al.*, 2009], moving away from the assumptions of full information sharing to analyze different levels of cooperation between agents [*Giuliani and Castelletti*, 2013]. We suggest this study shows traditional mathematical programming as used typically in hydro-economic models [*Harou et al.*, 2009] can be useful in simulating water markets. The assumption is that those transactions which generate the most regional benefits would also likely be attractive to individual profit-maximizing agents. If the analyst were to think this was not the case for a particular buyer or seller, or for a specific pair of these, special rules or customized transaction costs can be added to reflect this. The proposed framework is partially backed by economic theory in that under a market with perfect information those transactions that most benefit individuals concurrently most benefit the region. While a river basin will never be a perfect market, the online trading bulletin boards and preapproved trades being considered by the Environment Agency of England and Wales mean these assumptions may be close enough for model results to be informative and help policy-makers assess system performance.

We see this contribution as a step toward the goal of simulating markets. Our goal is to partially address the omissions of past hydro-economic modeling efforts meant to evaluate water trading systems as noted by *Griffin* [2006] (see section 1). In the proposed approach, individual trade transactions are tracked, detailed spatially and temporally specific transaction costs can be assigned, and abstractor behaviors are represented by constraint equations. More sophisticated behavioral models, perhaps borrowing from game theory [*Lejano and Davos*, 1995; *Madani*, 2010; *Mahjouri and Ardestani*, 2010; *Özelkan and Duckstein*, 1996; *Rogers*, 1969; *Wang et al.*, 2008] or multiagent system modeling theory [*Barreteau and Bousquet*, 2000; *Berger*, 2001; *Bonabeau*, 2002; *Feuillette et al.*, 2003; *Schreinemachers and Berger*, 2011; *van Oel et al*., 2010; *Yang et al.*, 2009] could potentially improve the behavioral component of this water market model. Currently agents make decisions each time step with minimal consideration of decisions made in previous time steps and others likely to occur in the future. Adding strategic decision points, like crop choice or infrastructure investment, at seasonal or annual scale could be appropriate extensions. The ability of agents to abstract water strategically across multiple licenses could also be added. In our case, this was done crudely using rules which for example blocked PWS from selling water if water use restrictions are in place due to low reservoir levels.

## 6. Conclusions

This paper described a generalized optimization model built to simulate water reallocation in a surface water spot market where downstream trades are preapproved by an environmental regulator. The model uses a node-arc multicommodity formulation that tracks individual seller-buyer transactions throughout a water resource network. The model's core driver is economic welfare maximization where demand curves are used to identify trades that generate most benefits. Transaction costs, definable for each buyer-seller pair, reflect costs incurred from pair-wise water trades. Abstractor-specific water management practices and plausible trading behaviors are represented as rules using constraints. The model was applied to the Great Ouse river basin in Eastern England which has 94 active public water supply, energy sector, industrial and agricultural surface water right holders. Current water management rules and behaviors including historical abstractions, environmental restrictions, and potential stakeholder trading behaviors are included. The water resource system is modeled with a weekly time step for a normal and dry historical year. A no-trading scenario is compared with the modeled trading to assess the potential hydrological and economic outcomes of the simulated water market. Results showed buyers favor sellers who can sell larger volumes to minimize the number of transactions. The basin's energy and agricultural licenses have the strictest environmental restrictions and benefit most from trades (68% and 8% increase in economic benefits from water use for the normal year and 81% and 67% in the dry year, for energy and agriculture, respectively). The primary model application limitations include the model's assumptions of perfect market information, the inclusion of only simplified longer-term planning, and the nonconsideration of strategic behavior of abstractors with multiple licenses. Groundwater use is not included in the model, but given the basin's groundwater resources are overabstracted, it would likely not change much under a surface water market.

## Appendix A: Nomenclature

### Abbreviations

**HoF**: Scenario where Hands-off Flow conditions are applied in which users can no longer abstract or trade water.
***junction***: No-demand and nonstorage nodes which join two or more links in the network.
***User***: The set of all licensed river abstractors including Agriculture, Industry, Water supply, and Energy.
***Owner***: The set of all water right holders, reservoirs, and the river.
Decision variable, the water flowing from node *i* to *j* with license holder k.
*inFl*_*i*_: External hydrological inflow at junction node *i*.
*CO*_*ij*_: Connectivity matrix which contains 1 if node *i* is connected to node *j*, 0 if no connection.
Reservoir *j* storage carried over from previous time step with water license *k*.
Reservoir *j* storage with water license *k*.
*tRes*_*j*_: Reservoir *j* target.
Water consumed by user *i* which is either bought from owner *k* or abstracted from river using user *i*'s license.
Water license leased for one time step by user *i*.
*ReturnFlow*_*ij*_: Water returned back to the river at downstream junction node *j* of user *i* based on the consumption factor of user *i*.
*DW*_*ij*_: Junction node *j* downstream of user *i*.
*consFactor*_*i*_: Fraction of water evaporated relative to diverted for user *i*.
*Discharge*_*j*_: Discharge sink *j* at the mouth of the river.
*WKLi*_*i*_: Weekly license allowance for user *i* to abstract water from river.
*YrLi*_*i*_: Yearly license allowance for user *i* to abstract water from river.
*Penalty*_*j*_: Deviation of reservoir *j* from its target storage volume.
*flGA*_*j*_: Flow at gauge *j*.
*AlGA*_*j*_: Allowable flow at gauge *j*.
*RuGA*_*ij*_: Information with regards to the hands of flow condition which equals one if user *i* abstraction is controlled with the level of flow at gauge *j*.
*Q95GA*_*j*_: Q95 flow level at gauge *j*.
*UpGA*_*ij*_: Agriculture user *i* upstream of gauge *j*.
Water used by user *i* at time t including the abstraction and trading.
Selling limit for user *i* at time *t*.
*EWN*_*i*_: Historical expectation of water needs for user *i*.
*totalBenefit*_*i*_: Total benefit for user *i*.
*totalCost*_*ik*_: Cost of trading incurred for user *i* purchased from user *k*.
Variable costs for user *i* purchased from user *k*.
Fixed per trade costs for user *i* purchased from user *k*.

## Erratum

In the first sentence of the second paragraph of the Introduction, the words “The design of flexible water markets to perform at the highest level” have been replaced with the words “Modeling water markets.”
